# Down-regulation of estrogen receptor-alpha and rearranged during transfection tyrosine kinase is associated with withaferin a-induced apoptosis in MCF-7 breast cancer cells

**DOI:** 10.1186/1472-6882-11-84

**Published:** 2011-10-06

**Authors:** Xuan Zhang, Ridhwi Mukerji, Abbas K Samadi, Mark S Cohen

**Affiliations:** 1 Department of Surgery, University of Kansas School of Medicine, Kansas City, Kansas, USA

## Abstract

**Background:**

Withaferin A (WA), a naturally occurring withanolide, induces apoptosis in both estrogen-responsive MCF-7 and estrogen-independent MDA-MB-231 breast cancer cell lines with higher sensitivity in MCF-7 cells, but the underlying mechanisms are not well defined. The purpose of this study was to determine the anti-cancer effects of WA in MCF-7 breast cancer cells and explore alterations in estrogen receptor alpha (ERα) and its associated molecules *in vitro *as novel mechanisms of WA action.

**Methods:**

The effects of WA on MCF-7 viability and proliferation were evaluated by 3-(4, 5-dimethylthiazol-2-yl)-5-(3-carboxymethoxyphenyl)-2-(4-sulfophenyl)-2H-tetrazolium (MTS) assay and trypan blue exclusion assays. Apoptosis was evaluated by Annexin V-fluorescein isothiocyanate (FITC)/propidium iodide (PI) flow cytometry and Western blot analysis of poly (ADP-ribose) polymerase (PARP) cleavage. Cell cycle effects were analyzed by PI flow cytometry. Western blotting was also conducted to examine alterations in the expression of ERα and pathways that are associated with ERα function.

**Results:**

WA resulted in growth inhibition and decreased viability in MCF-7 cells with an IC50 of 576 nM for 72 h. It also caused a dose- and time-dependent apoptosis and G2/M cell cycle arrest. WA-induced apoptosis was associated with down-regulation of ERα, REarranged during Transfection (RET) tyrosine kinase, and heat shock factor-1 (HSF1), as well as up-regulation of phosphorylated p38 mitogen-activated protein kinase (phospho-p38 MAPK), p53 and p21 protein expression. Co-treatment with protein synthesis inhibitor cycloheximide or proteasome inhibitor MG132 revealed that depletion of ERα by WA is post-translational, due to proteasome-dependent ERα degradation.

**Conclusions:**

Taken together, down-regulation of ERα, RET, HSF1 and up-regulation of phospho-p38 MAPK, p53, p21 are involved in the pro-apoptotic and growth-inhibitory effects of WA in MCF-7 breast cancer cells *in vitro*. Down-regulation of ERα protein levels by WA is caused by proteasome-dependent ERα degradation.

## Background

Breast cancer is the most common cancer and the second most common cause of cancer-related death among women in the United States [1]. This disease is usually treated through surgery and/or radiotherapy, supported by adjuvant endocrine or chemo-therapy. Unfortunately, most tumors acquire resistance during classical treatments [2]. Therefore, there is a need for developing novel therapeutics for breast cancer. Approximately 70% of breast cancers are estrogen receptor alpha (ERα)-positive [3]. ERα plays an important role in these cancers via both ligand-dependent and -independent mechanisms [4]. ERα is a member of the super family of nuclear receptors that function as transcription factors. In addition to estrogen-induced activation, it also interacts with growth factor pathways [5]. The role of ERα in breast cancer development has been extensively investigated. Transient over-expression of ERα promotes cell survival and estrogen-independent growth [6] whereas ERα knock-down induces cell apoptosis and growth inhibition [7] in estrogen-responsive MCF-7 breast cancer cells. Recent research also indicates that estrogen-independent ERα signaling and its interaction with growth factor receptors contribute to endocrine resistance in breast cancer treatment [8]. As such, ERα has become an important target in developing breast cancer therapies.

Withaferin A (WA) is a steroidal lactone occurring in *Withania somnifera *that has shown cytotoxicity in a variety of tumor cell lines and in animal cancer models *in vivo *without any noticeable systemic toxicity [9]. The mechanism of its action is currently under extensive investigation. It has been demonstrated that WA has the ability to alter numerous cancer-associated growth factor receptors, kinases, and transcription factors. It is a potent inhibitor of nuclear factor-κB activation [10], and angiogenesis [11]. In prostate cancer cell lines, WA binds to Heat shock protein 90 (Hsp90) and inhibits its chaperone activity, resulting in Hsp90 client protein degradation and tumor inhibition [12]. Recent research revealed that treatment with WA causes apoptosis and growth inhibition in both the ERα-negative, p53-mutant MDA-MB-231 and the ERα-positive, p53-wildtype MCF-7 breast cancer cell lines, but MCF-7 cells exhibit higher sensitivity to the apoptotic effect of WA [13,14]. The molecular mechanism underlying the anti-cancer effects of WA in breast cancer is not well defined.

We hypothesized that ERα and its associated molecular network such as REarranged during Transfection (RET) tyrosine kinase and p53 may be involved in the anti-cancer effects of WA in MCF-7 breast cancer cells. ERα and the tumor suppressor protein p53 exert opposing effects on breast cancer cell proliferation and apoptosis. ERα promotes proliferation of breast cancer cells whereas p53 induces growth inhibition and apoptosis [7]. RET is over-expressed in breast cancer ERα-positive breast cancer, and its activation stimulates MCF-7 breast cancer cell proliferation, survival and scattering [5,15]. In the present study, we examined the effects of WA on MCF-7 cell proliferation, viability, cell cycle distribution, and apoptosis, and addressed whether ERα and its associated molecular network may in part mediate the anti-cancer effects of WA in MCF-7 breast cancer cells.

## Methods

### Cell Lines and Reagents

The MCF-7 cells were purchased from the American Type Culture Collection (ATCC; Manassas, VA) and cultured in RPMI-1640 containing 10% fetal bovine serum (FBS), 100 units/Ml penicillin, 100 μg/mL streptomycin, 100 μg/mL gentamycin, 2 mM glutamine, and 1 mM pyruvate. The WA was purchased from ChromaDex (Irvine, CA). The antibodies against p53, RET, p21, poly(ADP-ribose) polymerase (PARP), caspase-3, caspase-7, p38 mitogen-activated protein kinase (p38 MAPK), phospho-p38 MAPK, heat shock factor 1 (HSF1) were purchased from Cell Signaling Technology (Danvers, MA) and the antibodies against ERα and survivin were purchased from Santa Cruz Biotechnology (Santa Cruz, CA). The anti-β-actin antibody was from Millipore (Billerica, MA). The anti-annexin V-fluorescein isothiocyanate (FITC)-conjugated and propidium iodide (PI) were from BD Bioscience (Rockville, MD).

### MTS assay

The effects of WA on MCF-7 cell viability was examined using 3-(4, 5-dimethylthiazol-2-yl)-5-(3-carboxymethoxyphenyl)-2-(4-sulfophenyl)-2H-tetrazolium (MTS) assay as previously described with minor modification [16]. Cells were seeded in 96-well microtiter plates (1.0 × 10^3 ^per well) in 100 μL of growth media. Cells were allowed to attach overnight and then treated with increasing concentrations of WA (0, 0.156, 0.313, 0.625, 1.25, 2.5, 5 μM) for 72 h. Cisplatin was used as a positive control. 20 μL of MTS solution was added at 72 h of treatment. After 3 h of incubation at 37°C (5% CO2), absorbance was measured at 490 nm with a microplate reader. The half-maximal inhibitory concentration (IC50) was obtained from the MTS viability curves using GraphPad Prism 5. Experiments were carried out in triplicates and the viability was expressed as the ratio of the number of viable cells with WA treatment to that without treatment. Experiments were repeated and similar results were obtained.

### Trypan Blue Exclusion Assay

Cell proliferation was evaluated using trypan blue exclusion assay as described before [17] with minor modification. MCF-7 cells were plated into 6-well plates at 20, 000 cells/well. After an overnight incubation, cells were treated with the vehicle (0.125% DMSO) or different doses of WA (0, 0.5, 1, and 2.5 μM) for 24 h. Cells exposed to 0.2% Trypan blue were then counted in a hemocytometer, and cells stained with Trypan blue were excluded. Percentage of viable cells was calculated based on the ratio of viable cell to total cell population in each well. The proliferation rate was calculated based on the number of viable cells in WA-treated group versus vehicle-treated group. Experiments were repeated to confirm the accuracy of the results.

### Annexin V-FITC/PI Flow Cytometry

The effects of WA on MCF-7 cell apoptosis was examine using annexin V-fluorescein isothiocyanate (FITC)/propidium iodide (PI) flow cytometry as described before [16]. Cell were treated with 0, 1, 2.5, 5 μM of WA for 24 h or with 2.5 μM of WA for 0, 6, 12 and 24 h. Detached and adherent cells were then collected and labeled for 15 min at room temperature with annexin V-FITC (1 μg/mL) and with PI (40 μg/mL) and immediately analyzed on a BD™LSRII flow cytometer (BD Biosciences) using BD FACSDiva6.0 software.

### Cell Cycle Analysis

The effects of WA on cell cycle distribution was examined using PI flow cytometry as described before [16] with minor modification. MCF-7 cells were seeded in complete medium (2 × 10^5 ^per 100-mm plate). After an overnight incubation, cells were treated with 0, 0.5, 1, 2.5 μM of WA for 24 h or with 2.5 μM of WA for 0, 6, 12 and 24 h. Detached and adherent cells were collected and fixed in 70% ethanol in phosphate-buffered saline (PBS) at -20°C overnight. Cells were then resuspended in PBS containing 40 μg/mL PI and 100 μg/mL RNAse and incubated for 30 min at 37°C in the dark. Samples were then analyzed on a on a BD™LSRII flow cytometer (BD Biosciences) using BD FACSDiva6.0 software.

### Western Analysis

MCF-7 cells were treated with 0, 0.5, 1, 2.5, 5 μM of WA for 24 h or with 2.5 μM of WA for 0, 1, 3, 6, 12, 24 and 48 h. Detached and adherent cells were collected, and total proteins were extracted using radioimmuno-precipitation assay buffer (RIPA buffer; 20 mM Tris [pH 7.5], 150 mM NaCl, 1% IGEPAL CA-630, 0.5% sodium deoxycholate, 1 mM ethylenediaminetetra-acetic acid, and 0.1% SDS) containing a protease/phosphatase inhibitor cocktail (0.1 mg/ml PMSF, 30 μl/ml of aprotinin, 5 μg/ml of leupeptin, and 1 mM sodium orthovanadate; Sigma). Protein concentrations were determined using the BCA protein assay reagent kit (Pierce, Rockford, IL). Equal amount of proteins were subjected to SDS-polyacrylamide gel electrophoresis (PAGE) and electroblotted onto nitrocellulose membranes (Hybond; Amersham, Piscataway, NJ). After blocking with 3% nonfat dry milk in PBS for 1 h at room temperature, blots were incubated with appropriate primary antibodies overnight at 4°C. Blots were then washed and incubated with appropriate horseradish peroxidase (HRP)-conjugated secondary antibodies for 1 h and protein bands were visualized using a chemiluminescence kit (Thermo Scientific, Rockford, IL). Next, blots were stripped and reprobed for β-actin. The expression level of each protein was normalized to the level of β-actin.

In order to examine whether WA down-regulates ERα protein at post-translational level, we treated MCF-7 cells with 100 μg/ml of protein synthesis inhibitor cycloheximide in the absence or presence of 2.5 μM WA for 1, 2, 4, or 6 h. Cells were collected at each time points and total proteins were isolated and subjected to Western analysis for ERα and β-actin.

To examine whether proteasomal degradation of ERα is involved in ERα protein down-regulation by WA, MCF-7 cells were pre-treated with 30 μM of proteasome inhibitor MG132 for 1 h before treatment with 2.5 μM WA for 6 h. Cells were then collected and proteins in triton-soluble or triton-insoluble fractions were isolated as described previously [18]. Briefly, cells were washed with PBS and lysed in RIPA buffer for 10 min on ice. After the cell suspensions were centrifuged at 16, 000 × *g *for 15 min at 4°C, the supernatants were collected as the Triton-soluble fraction. The pellet (Triton-insoluble fraction) was lysed in 2% SDS in 50 mM Tris-HCl and boiled for 15 min. The protein concentration of each sample was determined using the BCA protein assay. Samples were subjected to Western analysis for ERα and β-actin.

### Statistical Analysis

All data were analyzed using SPSS Version 17.0 software (SPSS, Inc.). ANOVA was used for comparison across treatment regimes. When an *F *test indicated statistical significance, post hoc analysis was made using the Tukey's honestly significant difference procedure. Significance was set at *P *< 0.05 for all comparisons.

## Results

### WA causes growth inhibition in MCF-7 cells

**Figure 1:**
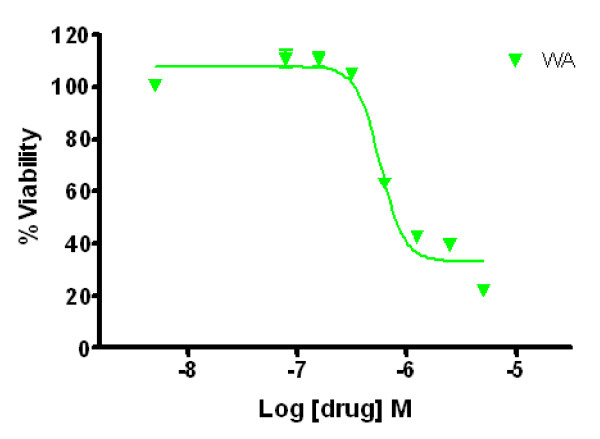
**Effect of WA on MCF-7 cell viability as measured by MTS assay**. Cells (1 × 10^3 ^per well) were cultured in 96-well plates for 24 h, and treated with indicated concentrations of WA for 72 h. The viable cells were quantified by MTS assay in triplicates.

**Figure 2:**
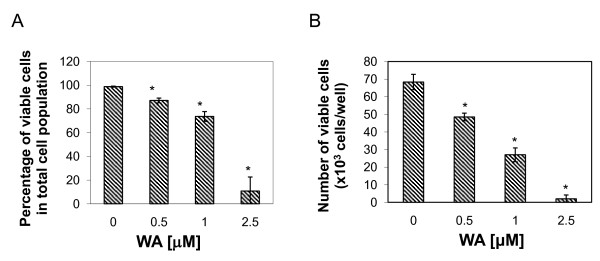
**Effect of WA on MCF-7 cell proliferation as measured by Trypan blue exclusion assay**. Cells were treated with DMSO or the indicated concentrations of WA for 24 h. A, percentage of viable cells in total cell population; B, the number of viable cells. Data were presented as mean (n = 3) ± SD. *, P < 0.05, significantly different from control by one-way ANOVA.

The growth-inhibitory effect of WA on MCF-7 cells was demonstrated by MTS assay (Figure 1) and trypan blue exclusion assay (Figure 2). As shown in Figure 1, treatments with WA for 72 h at concentrations between 313 nM and 5 μM reduced the number of viable cells. 50% inhibition of cell viability was induced with 576 nM of WA at 72 h. Similarly, Trypan blue exclusion assay showed that WA significantly decreased the number of viable MCF-7 cells at 24 h after treatment in a dose-dependent manner. WA decreased the percentage of viable cells in total cell population to 50% at between 1 to 2.5 μM, and decreased the number of viable cells to 50% compared to controls at between 0.5 to 1 μM.

### WA induces cell cycle arrest in MCF-7 cells

**Figure 3:**
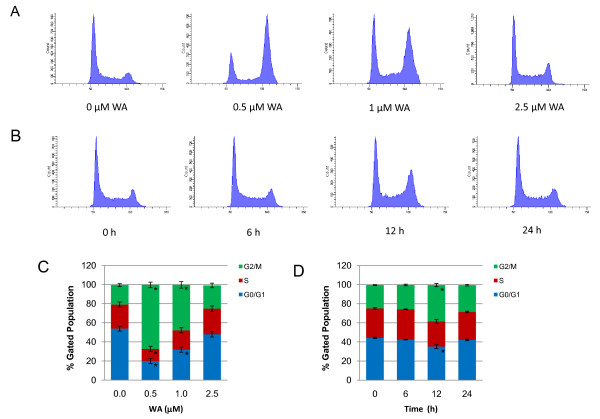
**Effect of WA on MCF-7 cell cycle distribution as measured by propidium iodide flow cytometry**. A and C, cells were treated with DMSO or the indicated concentrations of WA for 24 h. B and D, cells were treated with 2.5 μM WA for indicated time periods. Results were presented as mean (n = 3) ± SD. *, P < 0.05, significantly different from control by one-way ANOVA.

To examine whether the growth inhibitory effect of WA on MCF-7 cells was partly due to cell cycle arrest, we performed cell cycle analysis using PI staining. MCF-7 cells were treated with 0, 0.5, 1 and 2.5 μM of WA for 24 h or with 2.5 μM of WA for 0, 6, 12 and 24 h. As shown in Figure 3, treatment with WA induced G2/M cell cycle arrest in MCF-7 cells in a dose- and time-dependent manner. Interestingly, WA induced a decrease in G0/G1 fractions and an increase in G2/M fractions at 24 h, which were most evident with 0.5 μM of WA and gradually subsided with increasing doses. In the 2.5 μM WA time course study, the cells showed transient G2/M cell cycle arrest at 12 h. By 24 h, the percentage of cells in G2/M phase returned to control levels.

### WA induces apoptosis in MCF-7 cells

**Figure 4:**
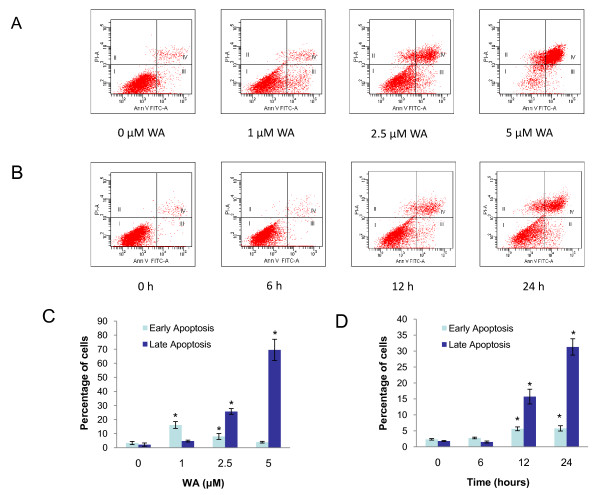
**Effect of WA on MCF-7 cell apoptosis as measured by Annexin V-FITC-propidium iodide flow cytometry**. A and C, cells were treated with DMSO or the indicated concentrations of WA for 24 h. B and D, cells were treated with 2.5 μM of WA for indicated time periods. Results were presented as mean (n = 3) ± SD. *, P < 0.05, significantly different from control by one-way ANOVA.

**Figure 5:**
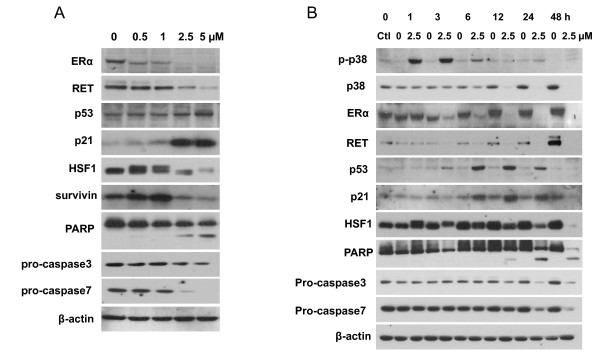
**Effect of WA on MCF-7 cell protein expression as measured by Western analysis**. A, Western analysis of the expression of ERα, RET, p53, p21, HSF-1, survivin, PARP, pro-caspase3, pro-caspase7 at 24 h after treatments with increasing concentrations of WA in MCF-7 cells. B, Western analysis of the expression of p38, phospho-p38, ERα, RET, p53, p21, HSF-1, survivin, PARP, pro-caspase3, pro-caspase7 at 0, 1, 3, 6, 12, 24, and 48 h after treatment with 2.5 μM of WA in MCF-7 cells.

To address whether WA causes apoptosis, we first did Annexin V-FITC/PI flow cytometry. As shown in Figure 4, WA significantly induces apoptosis in a dose- and time-dependent manner. In the dose response study, MCF-7 cells were treated with 0, 1, 2.5 and 5 μM of WA for 24 h. Early apoptosis (defined as Annexin V-positive/PI-negative) was induced by 1 μM WA at 24 h (12.7% increase compared to controls). 2.5 μM of WA induced more late apoptosis (23.5%) than early apoptosis (4.6%), while 5 μM of WA mainly induced late apoptosis (67.4%). In the time course study, cells were treated with 2.5 μM of WA for 0, 6, 12 and 24 h. No significant increase in apoptosis could be seen at 6 h. At 12 and 24 h, increase in both early apoptosis (3.3% and 3.4%, respectively,) and late apoptosis (13.9% and 29.5%, respectively) was induced. The WA-induced apoptosis in MCF-7 cells was also confirmed by Western analysis of PARP cleavage as well as caspase-3 and caspase-7 activation (Figure 5A and 5B). In the dose response study, 2.5 or 5 μM of WA significantly increased PARP cleavage at 24 h, which is associated with a decrease in pro-caspase3 and pro-caspase7 levels. In the time course study, slight but significant increase in PARP cleavage could be seen at 12 h after treatment with 2.5 μM of WA. By 24 h, PARP cleavage became more apparent.

### WA regulates ERα and its associated signaling pathways

To examine the molecular pathways underlying the anti-proliferative and pro-apoptotic effects of WA, we examined ERα protein levels and its associated signaling pathways by Western analysis (Figure 5A). WA markedly decreased ERα protein levels in a dose- and time-dependent manner. ERα protein levels diminished at 24 h after treatment with 2.5 or 5 μM of WA. The decrease in ERα occurred as early as 3 h post WA treatment (Figure 5B). We then examined the protein expression of RET kinase, which is over-expressed in a subset of ERα-positive breast cancers and is a novel proliferative pathway interacting with ERα signaling in MCF-7 breast cancer cells [5]. We found that WA caused a marked decrease in RET protein expression in parallel with ERα down-regulation.

Since ERα counteracts the action of the tumor suppresser p53, which is another major regulator of MCF-7 cell proliferation and survival, we next examined the effects of WA on p53 protein expression. We found that p53 was up-regulated by WA in MCF-7 cells, starting at 3 h and reaching maximum increase between 6 to 12 h post-treatment with 2.5 μM of WA. As p21 is a key inhibitor of cell cycle progression in MCF-7 cells, and is often stimulated by p53 [19], we analyzed the expression of p21 protein and correlated it with that of p53. We found that p21 expression was markedly up-regulated by WA as compared to controls, with kinetics closely following p53 expression (Figure 5B).

Survivin, a member of the inhibitors of apoptosis family, is a critical regulator of proliferation [20]. Because p53 is a known repressor of survivin expression and ERα inhibits this effect of p53, we decided to examine survivin expression after WA treatment in MCF-7 cells. Intriguingly, survivin protein expression was markedly decreased at 24 h after treatments with 2.5 or 5 μM of WA, while WA at 0.5 or 1 μM slightly increased survivin protein levels, suggesting other pathways that may control survivin expression. Since an earlier study reported that knockdown of HSF1 leads to a decrease of survivin expression [21], we examined the effect of WA on HSF1 protein expression. Interestingly, 2.5 or 5 μM of WA markedly decreased HSF1 expression at 24 h, while an additional band of HSF1 with slower mobility showed up after treatments with 0.5 or 1 μM of WA for 24 h, which likely indicates phosphorylation/activation of HSF1 (Figure 5A). The expression patterns of HSF1 and survivin appeared to correlate with each other.

We also examined the effect of WA on the expression and phosphorylation of the pro-apoptotic p-38 MAPK. We found that 2.5 μM of WA did not affect the levels of total p-38 MAPK protein expression within 3 h, but gradually decreased it after 6 h. In contrast, phospho-p38 MAPK was transiently increased by WA at 1 h and 3 h post treatment.

**Figure 6:**
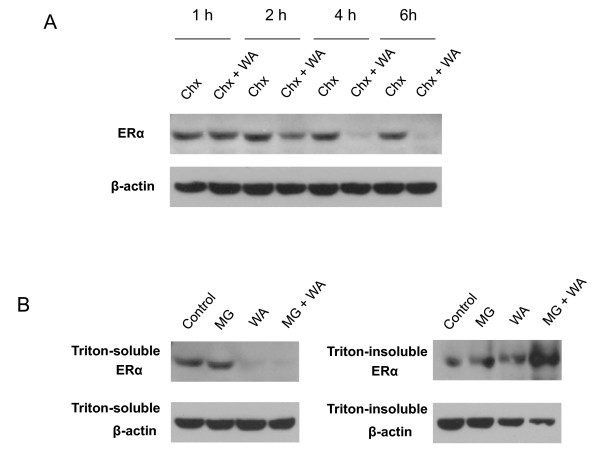
**WA down-regulates ERα expression at post-translational level by inducing proteasomal degradation of ERα protein**. A, Western analysis of the expression of ERα after treatment with 100 μg/ml cychloheximide (Chx) in the absence or presence of 2.5 μM WA for 1, 2, 4, or 6 h. B, Western analysis of the expression of ERα after treatment with 30 μM MG132 (MG) for 1 h followed by 2.5 μM WA for 6 h. Proteins from both triton-soluble and triton-insoluble fractions were examined.

To elucidate the mechanism of ERα protein down-regulation, protein synthesis inhibitor cycloheximide or proteasome inhibitor MG132 was used together with WA. We found that ERα protein levels became markedly lower at 2 h after treatment with cycloheximide plus WA as compared to cycloheximide alone, and even lower by 4 or 6 h (Figure 6A), indicating that WA post-translationally down-regulates ERα protein levels. In cells treated with MG231 and WA, the ERα protein was lost from Triton-insoluble fraction and accumulated in the Triton-insoluble fraction (Figure 6B), indicating that WA caused ERα protein aggregation and then induced proteasome-dependent ERα protein degradation.

## Discussion

In this study, we aimed to examine the anti-cancer effects of WA in ERα-positive MCF-7 breast cancer cells and explore the role of ERα and pathways associated with it in mediating these effects. We found that WA has potent inhibitory effects on the protein expression of ERα as well as RET tyrosine kinase that interacts with ERα pathway. WA also strongly up-regulated the protein levels of p53 and its down-stream target p21. Furthermore, WA at 2.5 or 5 μM markedly decreased HSF1 and survivin protein levels. These molecular changes correlate well with WA-induced dose- and time-dependent alterations in cell viability and apoptosis, suggesting that these are key factors involved in the anti-cancer effects of WA.

Most of breast cancer cases are hormone-dependent and higher levels estrogen are linked to increased risk of breast cancer [22]. ERα is a transcription factor that plays a key role in mediating estrogen signaling. Recent research revealed that ERα also plays a critical role in breast cancer progression via estrogen-independent mechanisms [23]. Knockdown of ERα expression using siRNA for ERα has been found to inhibit cell proliferation and induce apoptosis in MCF-7 breast cancer cells [7]. As such, ERα has become a molecular target in the development of therapeutics for breast cancer. In this study, WA treatment caused drastic decrease in ERα protein levels, which most likely contributed to growth inhibition and apoptosis.

RET is a receptor tyrosine kinase that mediates the proliferative and pro-survival effects of GDNF family growth factors in breast cancer. It is over-expressed in breast cancer, with higher levels in ERα-positive tumors as compared to ERα-negative tumors. Activation of RET stimulates MCF-7 breast cancer cell proliferation, survival and scattering [5,15]. RET pathway functionally interacts with ERα pathway [5]. In the present study, we found that WA down-regulates RET protein levels in parallel with ERα depletion. Down-regulation of both ERα and RET pathways may thus provide more effective anti-tumor activity.

The tumor suppressor p53 exerts its anti-proliferative action by inducing reversible or irreversible cell cycle arrest or apoptosis [24]. P53 can block cell cycle progression and/or induce apoptosis by transactivation of specific target genes, including p21Waf-1/Cip1 (p21), Gadd45, cyclin G and Bax [25,26]. Loss of p53 activity inhibits apoptosis and accelerates the appearance of tumors in transgenic mice [27]. The p53 gene is mutated in approximately 20% of breast cancers. Thus, although the majority of breast cancers have wild-type p53, its anti-cancer function remains suppressed. Additionally, p21 is a critical cell cycle inhibitor in many cancer cells including MCF-7 cells [28]. It has been found to be essential for G2/M cell cycle arrest upon DNA damage [29]. Although its role in apoptosis is controversial in general, it has been reported to induce apoptosis in MCF-7 cells [19,30]. In the present study, we found that WA increased the protein levels of p53 and p21, suggesting that they are involved in the growth inhibition and pro-apoptotic effects of WA. The pattern of p21 correlated well with that of p53, indicating that p53 may play a role in p21 up-regulation by WA. An up-regulation of phospho-p38 MAKP was observed at 1 h and 3 h post WA treatment, preceding the up-regulation of p53. Since phopho-p38 MAPK has been demonstrated to up-regulate p53 expression [31], and WA is able to induce apoptosis by activating p38 in leukemic cells [32], it appears that the p38 pathway may be an up-stream pathway involved in the anti-cancer effects of WA in MCF-7 cells.

Accumulating evidence indicates that there is a cross-talk between pathways mediated by ERα and p53. ERα and p53 exert opposing effects on breast cancer cell proliferation [7]. ERα binds directly to p53, leading to down-regulation of transcriptional activation or depression by p53. For example, wild-type p53 can be functionally inhibited by ERα leading to up-regulation of survivin and suppression of apoptosis [7]. RNA interference-mediated knockdown of ERα insulted in reduced survivin expression and enhanced apoptosis in MCF-7 breast cancer cells [7]. The depletion of ERα in combination with the up-regulation of p53 following WA treatment in MCF-7 cells may render the cells more sensitive to apoptosis. The fact that WA can also induce apoptosis, although not as effective, in MDA-MB-231 breast cancer cell line [[14], and unpublished data from our lab] which is ERα negative and p53-mutant, suggests that WA targets multiple pathways in its anti-cancer function, and neither ERα nor wild-type p53 is indispensable for the anti-cancer effect of WA. However, it is reasonable to postulate that in ERα-positive and p53-wild-type breast cancer cells, depletion of ERα and up-regulation of p53 substantially contribute to the anti-cancer effects of WA.

HSF1, the transcription factor for heat shock proteins (HSP), has recently been shown as a facilitator of transformation in breast cancer [33,21]. HSF1 inactivation inhibits the progression of a wide spectrum of cancers [34,35]. As the central regulator of HSP expression, HSF1 is also a target in designing anti-breast cancer therapies. HSF1 is another up-stream regulator of survivin. Down-regulation of HSF1 leads to decreased survivin expression in breast cancer cells [21]. In the present study, 2.5 or 5 μM of WA markedly decreased HSF1 and survivin expression at 24 h, suggesting that HSF1 may play a role in WA-induced apoptosis in part by regulating survivin expression. At lower concentrations or early time points, WA induced HSF1 phosphorylation, which is likely due to a transient defensive response of cells to the stress caused by WA.

In this study, WA induction of apoptosis in MCF-7 cells was confirmed by Annexin V assay as well as PARP cleavage and decreased expression of the pro-caspase3 and pro-caspase7. The amount of pro-caspase7 is much more abundant as compared to pro-caspase3 in MCF-7 cells, and this is in agreement with earlier reports [36]. At 1 μM, WA induced mainly early apoptosis. At higher concentrations (2.5 and 5 μM), the number of early apoptotic cells decreased whereas more late apoptotic cells were detected. It appears that whether it is early apoptosis or late apoptosis primarily depends on the concentration of WA, as mainly late apoptosis was detected with 2.5 μM WA at 12 h when apoptosis became detectable. WA causes minimal necrosis in MCF-7 cells. The small number of cells gated to the necrosis fraction after treatment with WA is most likely artifact caused by trypsinization, because when we tried Annexin V experiments at 36 h after 2.5 μM WA treatment without trypsinization (cells were all detached by 36 h), nearly 100% of cells were gated to the last apoptosis fraction (data not shown).

A recently published paper by Hahm et al. [37] also reported down-regulation of ERα and up-regulation of p53. However, they declared that WA-mediated decline in ERα protein level is caused by transcriptional repression, instead of proteasomal degradation. Our data showed that WA decreased ERα at post-translational level by inducing aggregation and proteasome-dependent degradation of ERα protein. The reason why they failed to see any effect of MG132 on WA-induced decline in ERα protein level is most likely that they only examined ERα protein in Triton-soluble fraction but not in Triton-insoluble fraction.

## Conclusions

In summary, we report here that WA induces apoptosis and growth inhibition in MCF-7 breast cancer cells at least in part through down-regulation of ERα, RET, HSF-1, as well as up-regulation of p53 and p21. WA depletes ERα levels by inducing proteasomal degradation of ERα protein. These findings help to explain the mechanisms underlying the anti-cancer effect of WA in ERα-positive, p53-wild type breast cancer, and reveals WA as a promising agent in breast cancer therapy.

## Competing interests

The authors declare that they have no competing interests.

## Authors' contributions

XZ conceived the study, carried out the experiments, and wrote the manuscript. RM and AKS assisted XZ in carrying out the experiments. MSC participated in designing the experiments. All authors read and approved the final manuscript.

## Pre-publication history

The pre-publication history for this paper can be accessed here:

http://www.biomedcentral.com/1472-6882/11/84/prepub
